# Heterotopic Ossification after Arthroscopic Elbow Release

**DOI:** 10.1111/os.12801

**Published:** 2020-11-16

**Authors:** Chao‐qun Yang, Jun‐sheng Hu, Jian‐guang Xu, Jiu‐zhou Lu

**Affiliations:** ^1^ Department of Hand Surgery Huashan Hospital, Fudan University Shanghai China; ^2^ Key Laboratory of Hand Reconstruction, Ministry of Health Shanghai China; ^3^ Shanghai Key Laboratory of Peripheral Nerve and Microsurgery Shanghai China; ^4^ Department of Hand Surgery Xuzhou Renci Hospital Xuzhou China; ^5^ School of Rehabilitation Science Shanghai University of Traditional Chinese Medicine Shanghai China

**Keywords:** Elbow arthroscopy, Heterotopic ossification, Stiff elbow

## Abstract

**Objectives:**

To evaluate the incidence and risk factors of heterotopic ossification (HO) after arthroscopic elbow release.

**Methods:**

The present study included 101 elbows, with arthroscopic release performed on 98 patients over the 5‐year period from November 2011 to December 2015. Patients were divided into three groups: group 1, with elbow arthritis, including 46 elbows in 43 patients; group 2, with posttraumatic extrinsic elbow stiffness (without intraarticular adhesion), including 23 elbows in 23 patients; and group 3, with intrinsic contractures (with intraarticular adhesion), including 32 elbows in 32 patients. Arthroscopic elbow release was performed under general anesthesia. For intrinsic stiffness, a radiofrequency device was applied to release intraarticular scar tissue and create work space, which was rarely necessary in groups 1 and 2. In the postoperative period, X‐rays and CT scans were assessed at follow up to determine if there was HO formation, which was diagnosed when new calcifications were identified. The functional recovery was evaluated by comparing the range of motion (ROM) and pain relief preoperativley and postoperatively in each group. Other complications were also assessed postoperatively.

**Results:**

The patients’ mean age was 38.6 years (range, 12–66), with 57 males and 41 females. Mean follow‐up was 21 months (range, 4–56). The active ROM and Mayo elbow performance index (MEPS) were improved from 93° ± 8.3° to 126° ± 12.4° (*P* < 0.05) and 71.4 ± 7.6 to 91.3 ± 8.7 (*P* < 0.001) in group 1, 66° ± 10.3° to 121° ± 10.7° (*P* < 0.005) and 65.6 ± 9.2 to 93.5 ± 11.2 (*P* < 0.05) in group 2, and 46° ± 6.7° to 91° ± 11.1° (*P* < 0.001) and 52.3 ± 6.4 to 80.6 ± 9.4 (*P* < 0.005) in group 3. HO developed in 25/101 cases (25%) and 4 patients with severe cases underwent repeat surgery. Those in group 1 were primarily arthritis patients; there were 3 out 46 cases with minor HO evident on X‐ray. In group 2, 1/23 had minor HO. In group 3, 21/32 patients had HO; 4 cases were considered severe, 4 were considered moderate, and 13 were considered minor. The average flexion–extension arc was improved by 47° at the last follow up. Other postoperative complications included 8 cases of prolonged drainage from portal sites, 17 transient nerve palsies, 1 permanent radial nerve injury, and 1 patient who developed delayed‐onset ulnar neuritis. This patient was fully recovered 5 months after surgery.

**Conclusions:**

The high incidence of HO formation after arthroscopic elbow release may relate to improper application of a radiofrequency device. Minimizing thermal injury from these radiofrequency devices could reduce HO formation and improve postoperative functional recovery.

## Graphical abstract

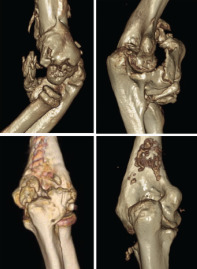

The occurrence of heterotopic ossification(HO) after arthroscopic elbow release is unexpectedly high in our practice. Suspicious risk factors relate to these HO formation was investigated in this study.

## Introduction

Arthroscopic elbow release has recently become a well‐accepted surgical technique for treating arthritis and posttraumatic stiffness^1^, ^2^, ^3^, ^4^, ^5^, ^6^. This technique is minimally invasive but relatively complicated, with more complications than open surgery. There are various complications during and after elbow arthroscopic surgery, including vascular injury, instrument breakage, compartment syndromes, septic arthritis, superficial infection, hematomas, persistent drainage from portal sites, and, most frequently, nerve injuries (transient and permanent)^7^, ^8^, ^9^, ^10^. We have benefited from previous studies on the potential risks and specific techniques for elbow arthroscopic surgery^11^, ^12^, ^13^, and the abovementioned complications during arthroscopic elbow release are not common in our practice. However, the occurrence of heterotopic ossification (HO) after arthroscopic elbow release, especially for posttraumatic stiffness, is unexpectedly high.

Heterotopic ossification refers to the formation of pathologic bone in nonosseous tissues. The common risk factors for HO formation include direct trauma, nueroaxis injury, full‐thickness burns, deep local infection, passive joint manipulation, microtrauma to the musculotendinous apparatus, and circulatory stasis^14^, ^15^, ^16^, ^17^, ^18^, ^19^, ^20^. Comparatively, the incidence of clinically significant HO in the elbow joint is more common than in other joints. Elbow stiffness with HO around the joint is very common after severe trauma (direct trauma, brain, or spinal injury). HO formation is also well known as a very challenging complication after major elbow surgery, especially after open elbow release. As new HO formation after open elbow release is one of the main problems that affects postoperative recovery, numerous studies have been done to clarify the mechanism. Although several prophylactic strategies, such as nonsteroidal antiinflammatory drugs (NSAID), bisphosphonates and even radiation therapy, have shown some effectiveness in preventing HO formation after surgery, HO formation as a complication of elbow surgery remains problematic.

While many studies have investigated HO formation after open elbow surgery, there are few case reports describing the HO formation after arthroscopic elbow surgery^21^, ^22^, ^23^. In these studies, HO formation after elbow arthroscopy was reported to be uncommon. However, these arthroscopic procedures were performed mainly on lateral epicondylitis or other sports injuries. HO formation rarely occurs in these injuries or diseases. Low incidence of HO formation after elbow arthroscopy in these procedures does not reflect the real relationship between HO formation and arthroscopic elbow surgery. There is a lack of published data on the incidence of HO formation after arthroscopic elbow release or other major elbow surgery.

Arthroscopic release can be performed for different types of elbow stiffness, including arthritic and posttraumatic stiffness (extrinsic or intrinsic). In our practice, the postoperative HO formation among different types of elbow stiffness is different. While HO formation in arthritic or extrinsic elbow stiffness is rare, there are very high rates of HO formation for intrinsic stiffness.

Functional recovery after elbow release is generally predictable if there is no HO formation. Elbow release using the arthroscopic technique is more promising as this technique results in less trauma and more rapid recovery. Therefore, prevention of HO formation after arthroscopic elbow release can greatly improve the functional recovery in patients with posttraumatic stiffness.

Although the true mechanism of HO formation after elbow surgery remains unclear, recognizing risk factors related to HO formation is helpful. Reviewing cases with significant HO formation after surgery, we found very intensive application of a radiofrequency device during arthroscopic release. After a private consultation with Shawn O'Driscoll from Mayo Clinic, we assume that the high incidence of HO formation after arthroscopic release is associated with thermal injury resulting from improper application of a radiofrequency device. The purpose of this study is: (i) to investigate the incidence of HO formation after arthroscopic release among different type of elbow stiffness; (ii) to evaluate the risk factors for HO formation after arthroscopic elbow release; and (iii) to modify the surgical technique to prevent HO formation after surgery.

## Methods and Patients

### *Inclusion and Exclusion Criteria*

Inclusion criteria were as follows: (i) stiff elbow consisting of elbow arthritis and posttraumatic stiffness; (ii) patients having undergone arthroscopic elbow release in the hand surgery department of Huashan Hospital from November 2011 to December 2015; (iii) preoperative HO around the elbow; (iv) incidence of HO formation after surgery among different groups; and (v) a retrospective study.

Exclusion criteria: (i) patients whose medical history was incomplete; (ii) patients who underwent other surgical interventions during the follow‐up period; (iii) patients who had not abided by medical advice to perform postoperative rehabilitation and exercise; and (iv) patients who had explicitly requested not to participate in the clinical research.

### *Group Allocations*

There were 101 elbows included, with arthroscopic release performed on 98 patients over the 5‐year period from November 2011 to December 2015. They were divided into three groups. Group 1 includes patients with elbow arthritis, with 46 elbows in 43 patients. This group presented with primarily arthritic symptoms and had only minor to moderate contractures. Group 2 includes patients with posttraumatic extrinsic elbow stiffness, with 23 elbows in 23 patients. This group of patients had contracture of the joint capsule and was made up of mostly forearm and humeral fractures. Group 3 included patients with posttraumatic intrinsic stiffness, with 32 elbows in 32 patients. This group was made up of mostly intraarticular fractures.

### *Surgical Technique*

#### *Anesthesia and Position*

Under general anesthesia, the patient was positioned in lateral decubitus. The arm was supported on a padded holder. A tourniquet was placed on the upper arm.

#### *Approach and Exposure*

All bony landmarks and ulnar nerves as well as arthroscopic portals were palpated and marked. Following tourniquet inflation, the joint was insufflated with 10 to 25 mL of saline through the “soft spot” portal site. The majority of the time we started with the proximal anterior medial portal. We identified the capsular contracture and released the contracture. For group 1, we performed debridement and osteophyte resection using the shaver and burr with rare use of a radiofrequency device. For group 2, we performed capsule resection with basket forceps and, again, rarely used a radiofrequency device. In group 3, if the contracture was primarily posterior, then we modified our approach and started by using the posterior approach first. In these cases, we found the use of a radiofrequency device very useful to create space for visualization and release. We also found the use of a radiofrequency device very helpful in releasing muscle from the distal humerus.

### *Postoperative Rehabilitation*

Patients started physical therapy on the second day after surgery. We used continuous passive motion (CPM) and an ice compress while the patients stayed in hospital (3–5 days). Hinged splinter and CPM were applied post‐discharge. The therapy continued until the patient reached what was considered maximum improvement. For patients with preoperative HO or osteophytes, postoperative X‐rays or CT scans were obtained to confirm the bony removal.

### *Clinical Outcome Evaluation*

Follow‐up radiographic examinations were performed within the first 6 weeks. Further X‐rays were based on the patient's clinical symptoms. In patients that we suspected to have HO, we obtained a CT scan. To determine if there was postoperative HO, the preoperative and immediate postoperative X‐rays was compared to the follow‐up X‐rays and CT scans. Postoperative HO was diagnosed when significant new calcifications were identified. The functional recovery was evaluated by comparing the range of motion (ROM) and pain relief preoperatively and postoperatively in each group. Other complications were also assessed postoperatively.

#### *Mayo Elbow Performance Index*

The Mayo elbow performance score (MEPS) is a rating system designed for evaluating both the objective function and subjective features (pain, stability, ROM, and the ability to perform daily activities). A total score of greater than 90 is excellent, 75–89 is good, 60–74 is fair, and below 60 is poor.

### *Complications*

Potential complications during and after surgery include prolonged drainage from portal sites, heterotopic ossification, nerve injury, and infection.

### *Statistical Analysis*

All statistical analyses were performed using IBM SPSS statistics version 22.0 (SPSS, Chicago, IL, USA). The data were expressed as means ±SD. The motion of extension and flexion, ROM, and MEPS score between preoperative and final follow up were compared using the paired *t*‐test and the χ^2^‐test. *P* < 0.05 was considered statistically significant.

## Results

### *Postoperative Follow Up*

The mean age was 38.6 years (range, 12–66) and there were 57 male and 41 female patients. Mean follow‐up was 21 months (range, 4–56). Active ROM and MEPS were improved from 93° ± 8.3° to 126° ± 12.4° (*P* < 0.05) and 71.4 ± 7.6 to 91.3 ± 8.7 (*P* < 0.001) in group 1, 66° ± 10.3° to 121° ± 10.7° (*P* < 0.005) and 65.6 ± 9.2 to 93.5 ± 11.2 (*P* < 0.05) in group 2, 46° ± 6.7° to 91° ± 11.1° (*P* < 0.001) and 52.3 ± 6.4 to 80.6 ± 9.4 (*P* < 0.005) in group 3 (Table 1), respectively.

**TABLE 1:** Data on heterotopic ossification (HO) formation and functional recovery after arthroscopic release in different groups (mean±SD)

Group number	Type of stiffness	Age (years)	Total number of involved elbows	HO developed cases	Cases underwent repeat surgery	Preoperative ROM (°)	Preoperative MEPS	Postoperative ROM (°)	Postoperative MEPS
1	Elbow arthritis	49.8 ± 5.4 (28–66)	46	3	0	93 ± 8.3	71.4 ± 7.6	126 ± 12.4	91.3 ± 8.7
2	Posttraumatic extrinsic elbow stiffness (without intraarticular adhesion)	33.1 ± 6.2 (12–51)	23	1	0	66 ± 10.3	65.6 ± 9.2	121 ± 10.7	93.5 ± 11.2
3	Intrinsic contractures (with intraarticular adhesion)	34.2 ± 7.3 (22–54)	32	21	4	46 ± 6.7	52.3 ± 6.4	91 ± 11.1	80.6 ± 9.4

MEPS, Mayo elbow performance index; ROM, range of motion.

### *Heterotopic Ossification Formation Rate after Surgery*

Heterotopic ossification developed in 25/101 cases (25%) and 4 patients with severe cases underwent repeat surgery. Group 1 were primarily arthritis patients; 3 out 46 cases had minor HO at postoperative follow up. Group 2 were patients with extrinsic stiffness, and there was 1 minor HO among 23 patients. Group 3 were patients with intrinsic stiffness, and 21 out of 32 patients showed HO at postoperative follow up. Out of the 21 cases, 8 were considered severe to moderate, and 13 were minor. The rate of HO formation was 6.5% in group 1 and 4.3% in group 2, while the HO formation rate in group 3 was 65.6%, which is significantly higher than in the former groups (*P* < 0.001).

### *Complications*

Postoperative complication included 8 cases of prolonged drainage from portal sites and 17 transient nerve palsies. These complications were all resolved without sequelae. There is 1 patient with a permanent radial nerve injury in group 3 who underwent nerve graft 4 months after primary arthroscopic surgery. One patient in group 2 developed delayed‐onset ulnar neuritis 2 weeks after surgery. She was fully recovered 5 months after surgery.

## Discussion

### *Incidence of Heterotopic Ossification Formation Associated with the Etiology*

This study showed that the incidence of HO after arthroscopic elbow release is relatively high, especially in patients with posttraumatic intrinsic elbow stiffness (21 out of 32 cases). Approximately 20% of patients have clinically relevant HO, which is associated with significant limitation in ROM. In the other two groups, which included patients with arthritis and extrinsic posttraumatic stiffness, respectively, HO formation was relatively low.

What makes the difference among the different groups? First, the type of stiffness varies in each group. Group 2 includes patients with extrinsic stiffness and group 3 with intrinsic stiffness. Does the type of stiffness lead to a different incidence of HO formation after surgery? In general, a patient with a traumatic stiff elbow is more likely to have HO after surgery. Previous trauma may contribute to HO, especially when there is nerve injury. In the literature, HO after open elbow release is approximately 6%. In this study, HO after arthroscopic release was present in 21 out of 32 patients in group 3. Therefore, the high incidence of HO formation in group 3 after arthroscopic release may be due to specific surgical techniques.

### *Suspicious Risk Factors during Arthroscopic Release Relate to Heterotopic Ossification Formation*

The basic surgical technique we applied for arthroscopic elbow release in different groups was the same. The main difference in group 3 was the aggressive application of the radiofrequency device during surgery. Group 3 includes patients with intrinsic elbow stiffness, characterized by scar tissue and small joint space. The radiofrequency device is efficient in releasing severe scar tissue and creating space for intrinsic stiff elbows. However, the radial frequency device produces a substantial quantity of heat if it is used in a very limited space without sufficient inflow. The temperature becomes very high in the surrounding tissue, and the thermal injury may lead to HO formation. We were not able to measure the temperature during surgery. However, indirect evidence indicated thermal injury to the joint. The smell and heat were obvious when using the radiofrequency device. The surrounding soft tissue was burnt after using the radiofrequency device. We observed a very high incidence of HO in these cases. In contrast, patients in groups 1 and 2, who rarely needed the radiofrequency device, demonstrated a very low incidence of HO.

**Fig 1:**
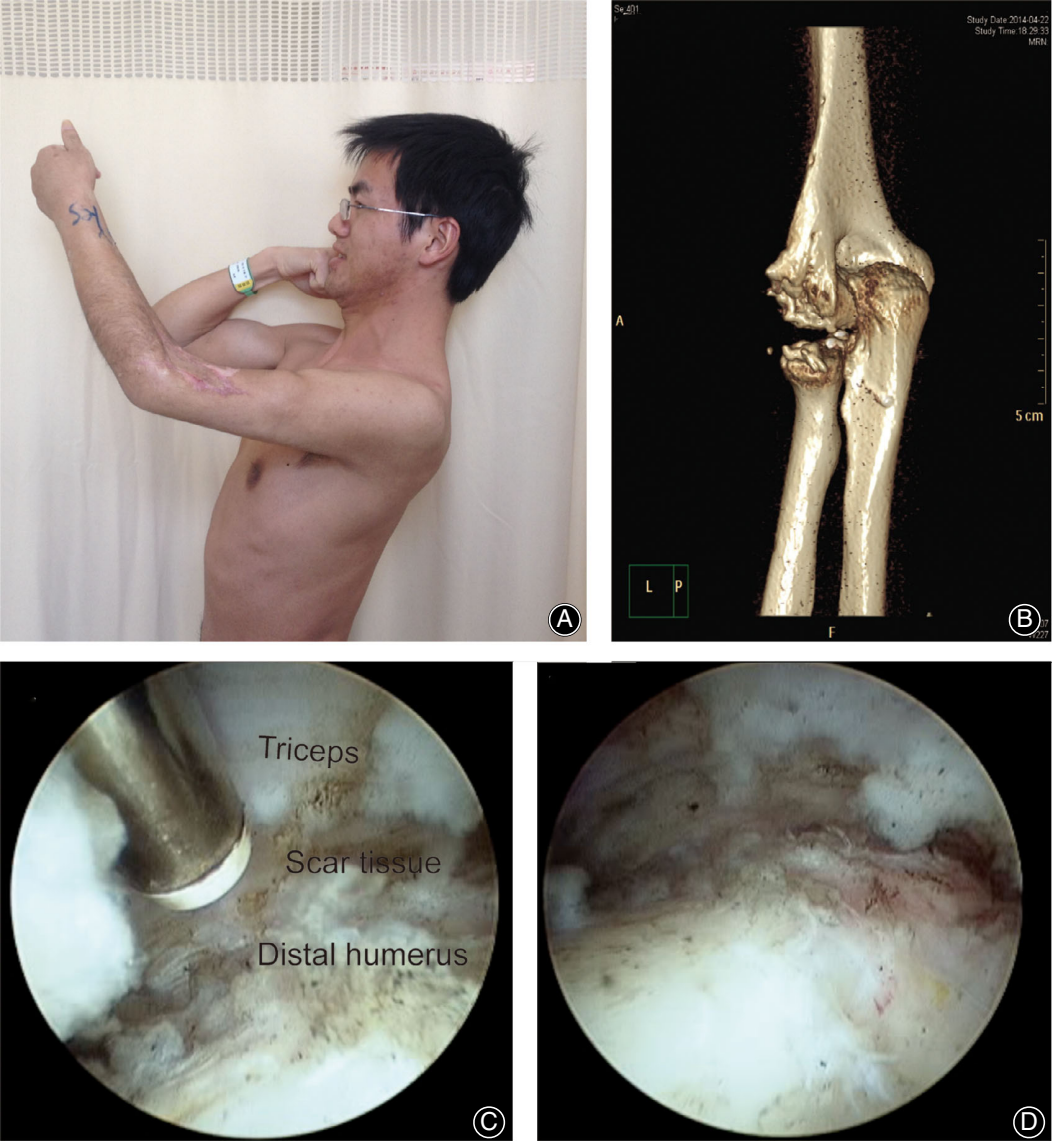
Posttraumatic stiff elbow in a 21‐year‐old man. (A) Preoperative physical examination shows very limited elbow flexion. (B) CT scan shows heterotopic ossification (HO) formation after previous surgery. (C, D) Radiofrequency was used to release muscle from the distal humerus.

**Fig 2:**
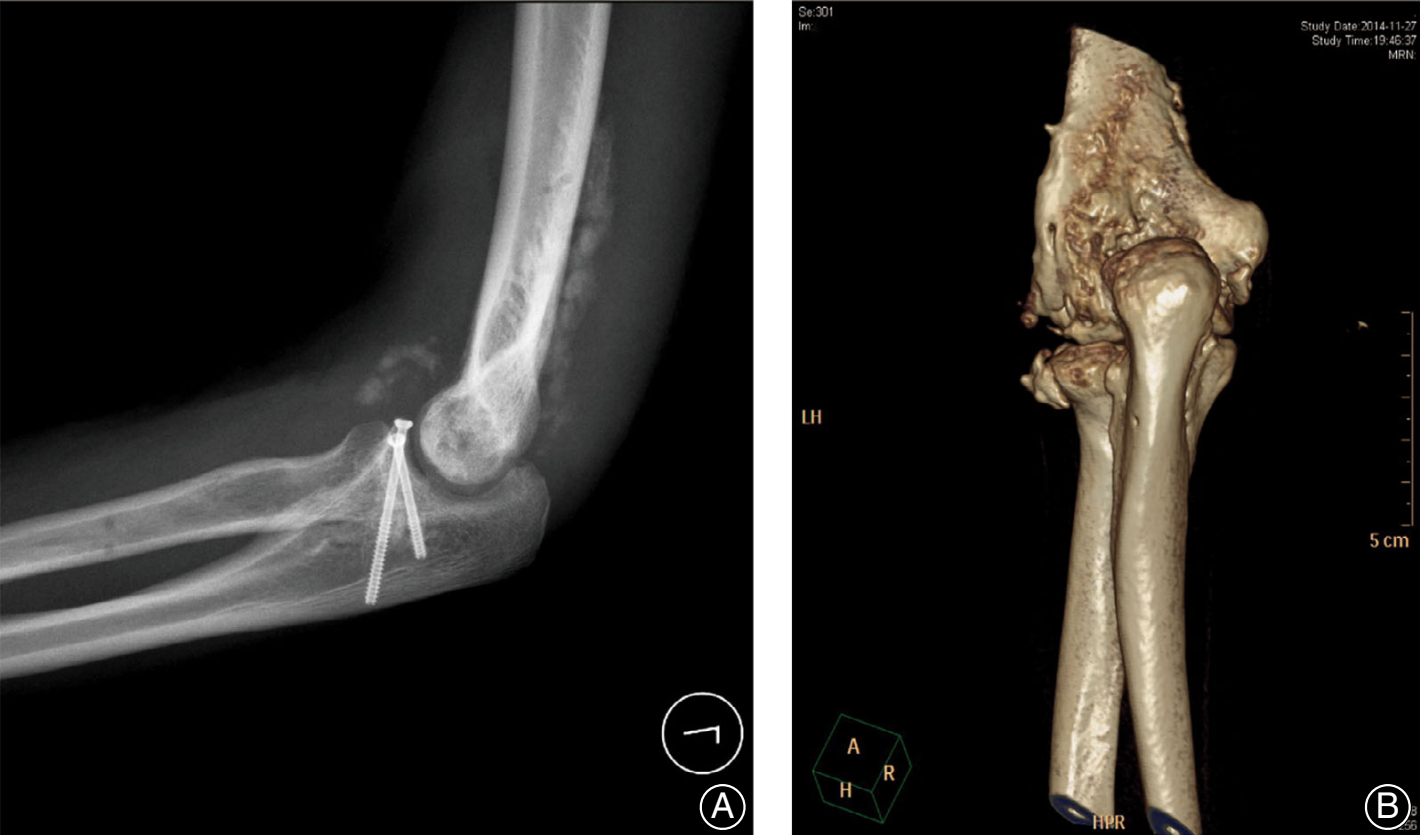
(A) Postoperative X‐ray shows heterotopic ossification (HO) formation at 1‐month follow‐up. (B) CT scan shows HO 6 months after surgery.

**Fig 3:**
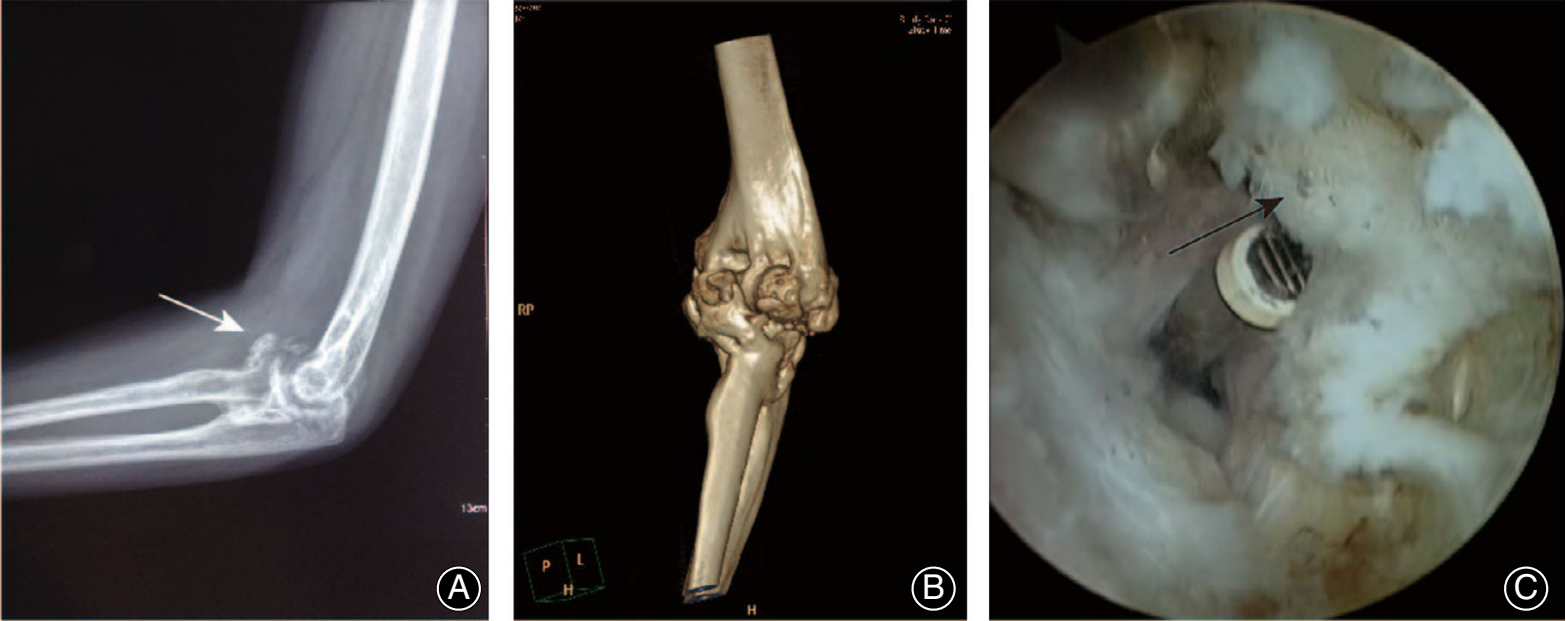
Posttraumatic stiffness with heterotopic ossification (HO) formation in a 56‐year‐old woman. (A, B) Preoperative X‐ray and CT scan shows HO in the front joint. (C) Radiofrequency was used to release the scars around HO.

**Fig 4:**
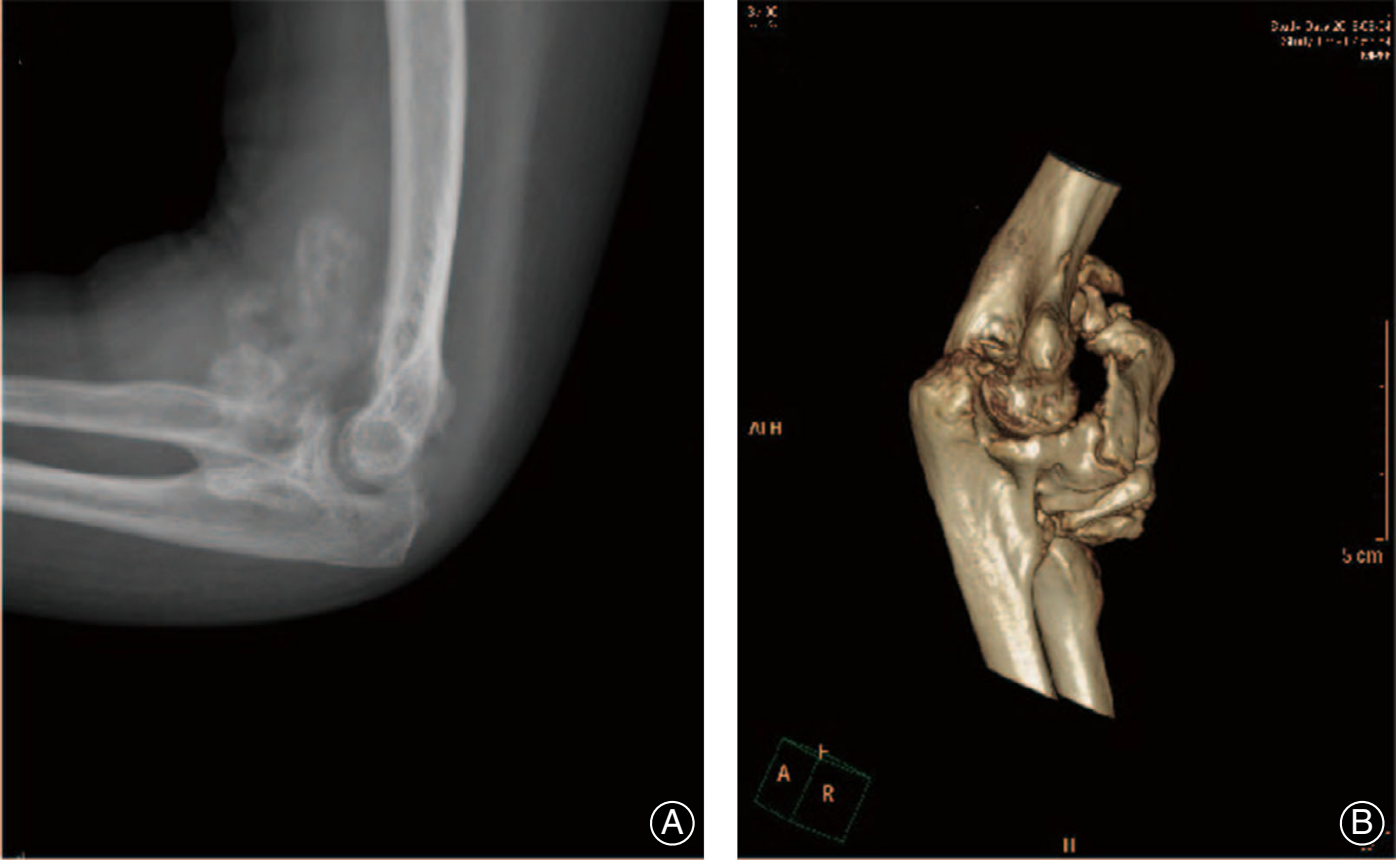
Heterotopic ossification (HO) formation after arthroscopic release. (A) X‐ray shows new HO formation at 5 weeks follow‐up. (B) CT scan shows mature HO at 8 months.

More straightforward evidence to prove the relationship between the application of radiofrequency and HO formation is the location of the HO. The HO usually occurred in the posterior compartment, as well as in the interval between the triceps and the humerus (Figs. 1, 2). We often released scar tissue with radiofrequency in those compartments or intervals as there were no major neurovascular structures. These HO were very likely to have resulted from intraoperative thermal injury due to radiofrequency application (Figs. 3, 4).

The association between thermal injury and HO formation has been widely reported^19^, ^20^, ^21^. However, the true mechanism remains unclear. Animal studies have also revealed the association between unmyelinated nerve injury and complex regional pain syndrome (CRPS), which is a variation of HO^24^, ^25^. As the radio frequency device apparently causes thermal injury to the surrounding unmyelinated nerve, it is reasonable to suppose that improper application of this device could lead to HO formation after surgery.

### *Prevention of Heterotopic Ossification Formation after Arthroscopic Elbow Release*

Preventing HO formation after arthroscopic elbow release is essential for achieving a good clinical result. The key point is to avoid using radiofrequency when there is no working space. Adequate space should be created by other instruments before using thermal devices. In addition, sufficient inflow needs to be maintained when using these devices. Also important is minimizing the usage of thermal devices even if there is adequate working space. The thermal device was mainly used for releasing severe scar tissue rather than for normal structures. We saw very low HO occurrence after arthroscopic elbow release because we minimized the application of the radiofrequency device. This further demonstrated that HO formation after arthroscopic release was highly associated with thermal injury.

### *Limitations*

As we know, there are multiple risk factors contributing to HO formation, such as previous trauma and individual variation. Although the thermal injury from the radio frequency device explained the HO formation after surgery, this retrospective study was not able to quantify the association between the severity of thermal injury and HO formation. Another limitation of this study is the evaluation of the postoperative HO. There was no well accepted classification system to use and the degree of HO was mainly determined by size. Personal experience in this process may lead to bias.

In conclusion, HO formation is a potential risk after arthroscopic elbow release in patients with posttraumatic intrinsic elbow stiffness. The predominant factor for HO formation after surgery is the thermal injury due to improper application of the radial frequency device. Minimizing thermal injury from these devices may reduce HO formation and improve postoperative functional recovery.

## Disclosure

The authors declare no potential conflicts of interest with respect to the research, authorship, and/or publication of this article. This study was supported by the Shanghai Municipal Key Clinical Specialty (shslczdzk05601), the Limb Function Reconstruction Clinical Medicine Center (2017ZZ01006), the Foundation of Systemic Assessment of Elbow Dysfunction (SHDC12018130), and the Municipal Hospital Newly‐Developing Cutting‐Edge Technologies Joint Research Program of Shanghai Shenkang Hospital Development Center (No. SHDC12018130).
